# Uncovering the potential mechanism of Xue Fu Zhu Yu Decoction in the treatment of intracerebral hemorrhage

**DOI:** 10.1186/s12906-022-03577-2

**Published:** 2022-04-12

**Authors:** Dao-jin Xue, Zheng Zhen, Ke-xin Wang, Jia-lin Zhao, Yao Gao, Yu-peng Chen, You-bi Shen, Zi-zhuang Peng, Dao-gang Guan, Tao Huang

**Affiliations:** 1 The Second Affiliated Hospital of Guangzhou University of Chinese Medicine, Guangzhou, 510000 China; 2 The Second Clinical Medical School of Guangzhou University of Chinese Medicine, Guangzhou, 510000 China; 3 Neurosurgery Center, Guangdong Provincial Key Laboratory On Brain Function Repair and Regeneration, Department of Cerebrovascular Surgery, Engineering Technology Research Center of Education Ministry of China On Diagnosis and Treatment of Cerebrovascular Disease, Zhujiang Hospital, Southern Medical University, Guangzhou, Guangdong, 510000 China; 4 Department of Biochemistry and Molecular Biology, School of Basic Medical Sciences, Southern Medical University, Guangzhou, 510000 China; 5 Guangdong Provincial Key Laboratory of Single Cell Technology and Application, Guangdong Province, Guangzhou, 510000 China; 6 Department of Psychiatry, First Hospital/First Clinical Medical College of Shanxi Medical University, Taiyuan, 030001 China; 7 Department of Bioinformatics, School of Basic Medical Sciences, Southern Medical University, Guangzhou, 510000 China

**Keywords:** Chinese herbal medicine (CHM), Intracerebral Hemorrhage (ICH), Important gene network model, Mechanism, Integrated pharmacology

## Abstract

**Background:**

Chinese herbal medicine (CHM) is characterized by “multi- compounds, multi-targets and multi-pathway”, which has advanced benefits for preventing and treating complex diseases, but there still exists unsolved issues, mainly include unclear material basis and underlying mechanism of prescription. Integrated pharmacology is a hot cross research area based on system biology, mathematics and poly-pharmacology. It can systematically and comprehensively investigate the therapeutic reaction of compounds or drugs on pathogenic genes network, and is especially suitable for the study of complex CHM systems. Intracerebral Hemorrhage (ICH) is one of the main causes of death among Chinese residents, which is characterized with high mortality and high disability rate. In recent years, the treatment of ICH by CHM has been deeply researched. Xue Fu Zhu Yu Decoction (XFZYD), one of the commonly used prescriptions in treating ICH at clinic level, has not been clear about its mechanism.

**Methods:**

Here, we established a strategy, which based on compounds-targets, pathogenetic genes, network analysis and node importance calculation. Using this strategy, the core compounds group (CCG) of XFZYD was predicted and validated by in *vitro* experiments. The molecular mechanism of XFZYD in treating ICH was deduced based on CCG and their targets.

**Results:**

The results show that the CCG with 43 compounds predicted by this model is highly consistent with the corresponding Compound-Target (C-T) network in terms of gene coverage, enriched pathway coverage and accumulated contribution of key nodes at 89.49%, 88.72% and 90.11%, respectively, which confirmed the reliability and accuracy of the effective compound group optimization and mechanism speculation strategy proposed by us.

**Conclusions:**

Our strategy of optimizing the effective compound groups and inferring the mechanism provides a strategic reference for explaining the optimization and inferring the molecular mechanism of prescriptions in treating complex diseases of CHM.

## Introduction

ICH refers to the hemorrhage caused by spontaneous and non-traumatic cerebral vascular rupture, which is often located in the deep brain tissues such as basal nucleus, putamen, and thalamus, etc. It has the characteristics of acute onset, rapid change of disease condition, high mortality and disability rate, and is more common in middle-aged and elderly people. According to the statistical results, the prevalence rate of hemorrhagic stroke in China is 406.16/100,000, and the incidence rate is 26.34/100,000 [1]. The proportion of cerebral hemorrhage in stroke patients in China (24%) is higher than which in developed countries [2]. ICH is an important disease that seriously endangers the health and quality of life of middle-aged and elderly people. The main methods in treating ICH include drug therapy, surgical treatment, etc. Among them, controlling blood pressure is the most commonly treatment based on drugs. In the early stage of disease, the blood clots produced after bleeding will destroy the peripheral normal nerve cells, which will lead to the aggravation of neurological dysfunction such as hemiplegia and speech disorder. Therefore, how to promote hematoma absorption as soon as possible and reduce edema around the blood clots are a difficult point in conservative treatment.

In recent years, reports about treating ICH with CHM have gradually increased. For example, Buyang Huanwu Decoction can alleviate brain edema after ICH, reverse blood–brain barrier (BBB) damage and reduce nerve function damage [3]. Di Dang Tang can alleviate the nerve function injury and has better brain protection [4]. Xue Fu Zhu Yu Decoction (XFZYD) is the most widely used in treating ICH. Clinical study confirmed that XFZYD combined with western medicine has a significant clinical effect in treating ICH, which can improve the hypercoagulable state of blood in patients, thus promote the absorption of cerebral hematoma and reduce the neurological deficit [5]. Modern pharmacological experiments have also found that XFZYD can reduce the level of serum asymmetric dimethylarginine (ADMA) in atherosclerotic rats, thereby increasing the synthesis and secretion of NO, and further improving the pathological degree of atherosclerosis [6]. In addition, it can promote the secretion of vascular endothelial growth factor (VEGF) by human umbilical vein endothelial cells (HUVEC), suggesting that it may promote the proliferation of HUVEC by promoting the secretion of VEGF, thus playing a role in removing blood stasis and promoting new life [7].

The prescription contains 11 herbs: *Rehmannia glutinosa (Gaertn.)* (15 g), *Bupleurum chinense DC* (5 g), *Glycyrrhiza uralensis Fisch* (5 g), *Citrus aurantium L* (10 g). *Platycodon grandiflorus (Jacq.) A.DC* (7.5 g), *Achyranthes bidentata Blume*(15 g), *Prunus alleghaniensis Porter* (20 g), *Carthamus tinctorius* (15 g), *Ligusticum chuanxiong S.H.Qiu* (7.25 g), *Paeonia veitchii Lynch* (10 g), *Angelica sinensis (Oliv.)* (15 g). Among these herbs, *Prunus alleghaniensis Porter* breaks blood and moistens dryness, while *Carthamus tinctorius L* promotes blood circulation and removes blood stasis to relieve pain. *Paeonia veitchii Lynch* and *Ligusticum chuanxiong S.H.Qiu* help the monarch to dispel blood stasis and promote blood circulation. Achyranthes bidentata Blume can enhance blood circulation, clear channels, remove blood stasis and relieve pain, and lead blood downward. *Rehmannia glutinosa (Gaertn.)* and *Angelica sinensis (Oliv.) Diels* are usually used to nourish blood and nourish yin, clear heat and promote blood circulation; *Platycodon grandiflorus (Jacq.) A.DC.* and *Citrus aurantium L* can be used to disperse lung, relieve sore throat, eliminate phlegm and discharge pus; *Bupleurum chinense DC* can be used to liver stagnation and qi stagnation, chest and rib pain, *Glycyrrhiza uralensis Fisch* has the efficacy of relieving pain and harmonizing various drugs. XFZYD composed of these herbs has the effects of eliminating edema and improving local circulation, so it is widely used in the treatment of ICH. Although it has been reported, the mechanism is still unclear. How to find the key compounds to deduce the potential mechanism is the key step to understand the treatment of ICH by XFZYD.

At present, systems pharmacology is a product of interdisciplinary research, including chemical and structural biology, bioinformatics, computer technology and mathematics, and also covers a large number of experimental disciplines including research technologies from cells, tissues to organs. In the recent researches, systems pharmacology is widely used to explore the hidden mechanisms of CHM prescriptions on complex diseases. Such as, Wang Chun et al. built a contribution index model to analyze the molecular mechanism of Zhi-zhu Wan in treating Functional Dyspepsia [8], Wang Kexin et al. quantified the molecular mechanism of Danggui Sini decoction (DSD), Guizhi Fuzi decoction (GFD), and Huangqi Guizhi Wuwu Decoction (HGWD), in treating rheumatoid arthritis based on the network communities detection model of network pharmacology [9], Gao Yao et al. constructed optimization model based on network pharmacology to detect the molecular mechanisms of Lang Chuang Wan in treating systemic lupus erythematosus [10]. Increasing research evidence shows that systems pharmacology has the characteristics of integrity and synergy in interpreting the underlying mechanisms of CHM prescription on complex diseases. Prescriptions in CHM has multi-compounds, multi-targets, multi-pathways mode of action in treating complex diseases, which reflects the system, integrity and coordination of CHM, while the design idea of system pharmacology is consistent with these properties of prescriptions in the treatment process.

Systems pharmacology of CHM provides novel ideas and perspectives for the study of complex CHM system. By using systems pharmacology technology, the theories and methods of studying the active substances and combinations of CHM, identifying the targets of pharmacodynamics compounds, and the relationship between pharmacodynamic substances and diseases were studied, so as to establish the pharmacodynamics of CHM and the basic theory of CHM based on systematic level. For example, Liu et al. used network pharmacology to prove the action of Jinshui Huanxian Formula on Idiopathic Pulmonary Fibrosis [11]. He et al. used systematic pharmacology to analyze the synergistic mechanism of LiuWei DiHuang Pill in Type 2 Diabetes Mellitus [12].

In this study, a new systems pharmacology model was designed for capturing the key compounds and deducing the potential mechanism of XFZYD in treating ICH. This model is based on novel node importance calculation method, and integrates compound monomer structure and target information, protein–protein interaction (PPI) network response mode and pathogenic genes reported in literature, and systematically explores the key effective compounds in treating ICH with XFZYD. The mechanism of CCG was deduced by using the target protein response score(TPRS) model. The reliability of the model is further confirmed by the verification of functional coverage and *vitro* experiments, which would provide methodological reference and advice for the systematic analysis of CHM in treating other complex diseases.

## Materials and methods

### Constructing the weighted pathogenic gene–gene interactions of ICH

The PPI data was extracted from seven published database, include STRING [13], BioGRID, HPRD, Dip, Intact, Reactome and Mint. These data were merged to construct comprehensive PPI network. ICH-related genes were extracted from Genecard database, and genes with a relevance score higher than the average score were retained as highly pathogenic genes. These pathogenic genes were mapped into the PPI network to build the weighted pathogenic gene–gene interaction network of ICH. Then the pathogenic gene–gene interactions were visualized by Cytoscape software (Version 3.7.2).

### Collectting chemical compounds of XFZYD

All compounds of XFZYD were derived from three public herb medical data sources, TCMSP database [14], TCM integrated database [15] and TCM database@Taiwan [16]. The chemical properties of these compounds, include molecular weight (MW), oral bioavailability (OB), DL (drug-likeness) and Caco-2 permeability (Caco-2) were obtained from TCMSP. In addition, some compounds which were proved to with higher concentration in XFZYD in experiments were kept for next step analysis.

### Selectting potential active compounds of XFZYD based on ADME models

Three ADME-related modules (OB, DL, and Caco-2) were used to screen biologically active compounds. OB stands for the radio of the oral constant-dose ingredient or drug that can be delivered to the circulation system [17]. Higher OB usually is an important indicator to determine the drug-like property of biologically molecules as therapeutic drugs. The compounds with OB ≥ 30% were selected as active compounds for further analysis. The transport rates (nm/s) of compounds in Human intestinal cell line Caco-2 mono layers represent the intestinal epithelial permeability. The compounds with Caco-2 > -0.4 were selected as the active compounds, because the compounds with a Caco-2 value less than -0.4 were impermeable. Drug similarity helps optimize pharmacokinetics and drug properties, such as chemical stability and solubility. The “drug-like” level of 0.18 is usually used as a selection criterion for the “drug-like” compounds in the CHM and was used in this study.

### Targets prediction of active compounds

Three public databases, Similarity Ensemble Approach (SEA) [18], HitPick [19], and Swiss Target Prediction [20] were used in the targets prediction of active compounds in XFZYD. All chemical structures were converted into canonical SMILES by the software Open Babel toolkit (version 2.4.1).

### Constructing node importance calculation method

For calculating the importance of each node in the network, we constructed a node importance calculation method (BCR), in which, n represent the number of nodes in the network; s, v and t represent nodes in the network; V represents the collection of nodes within the network; |V| represents the number of nodes; ${\upsigma }_{\mathrm{vt}}$ represents the number of the shortest path between nodes v and t; ${\upsigma }_{\mathrm{vt }(\mathrm{s})}$ is the number of the shortest path passing through node s; C_(s)_ represents the genes which contains nodes; ${\Delta }_{\mathrm{C}(\mathrm{s})}$ is the maximum distance between gene C and other genes passing through node s; dist_(s, w)_ represents the length of a shortest path between nodes s and w; The dist_(s, w)_ is equal to infinite if C_(s)_ ≠ C_(w)_, and it makes methods of this category cannot be applied to networks with disconnected genes. ${\mathrm{BR}}_{\mathrm{S}}$ represents the relationship between node s and other nodes.

$${{\mathrm{BR}}_{\mathrm{S}}}_{ }= \sqrt{\left(\sum_{\mathrm{s }\ne \mathrm{v }\ne \mathrm{t }\in \mathrm{V}}^{ }\frac{{\upsigma }_{\mathrm{vt }(\mathrm{s})}}{{\upsigma }_{\mathrm{vt}}}\right) \times \left[\frac{\left|\mathrm{ V }\left( {\mathrm{C}}_{\left(\mathrm{s}\right)}\right) \right|}{\left|\mathrm{ V }\right|} \times \frac{\sum_{\mathrm{w}\in \mathrm{C}\left(\mathrm{s}\right)}\left( {\Delta }_{\mathrm{C}\left(\mathrm{s}\right)}+1-{\mathrm{dist}}_{\left(\mathrm{s},\mathrm{w}\right)}\right)}{\mathrm{max }\{{\mathrm{dist}}_{\left(\mathrm{s},\mathrm{w}\right)}:\mathrm{w }\in \mathrm{C}(\mathrm{s})\}}\right]}$$

There are n nodes in the network. After being quantized, BR was sorted from small to large, and was represented by a new variable G.

$$\mathrm{G}=\left[{\mathrm{G}}_{1}, {\mathrm{G}}_{2}, {\mathrm{G}}_{3}, \dots , {\mathrm{G}}_{\mathrm{n}}\right]= \left[{\mathrm{BR}}_{\mathrm{i}}, {\mathrm{BR}}_{\mathrm{i}+1}, {\mathrm{BR}}_{\mathrm{i}+2}, \dots , {\mathrm{BR}}_{\mathrm{i}+\mathrm{m}}\right] ,\mathrm{ x}\in \left[1,\mathrm{ n}\right]\mathrm{ and i}+\mathrm{m}=\mathrm{n}$$

The new variable P represented the nodes in the network. Each P responds to its unique G.

$$\mathrm{P }\in \left\{{\mathrm{P}}_{1}, {\mathrm{P}}_{2}, {\mathrm{P}}_{3}, \dots , {\mathrm{P}}_{\mathrm{n}}\right\} \rightleftharpoons \left[{\mathrm{G}}_{1}, {\mathrm{G}}_{2}, {\mathrm{G}}_{3}, \dots , {\mathrm{G}}_{\mathrm{n}}\right]$$

BCR represented the important nodes selected form all nodes in the network. N represented natural number.

$$\mathrm{BCR }\in \left\{{\mathrm{P}}_{\mathrm{r}}, \dots , {\mathrm{P}}_{(\mathrm{n}-2)},{\mathrm{P}}_{(\mathrm{n}-1)},{\mathrm{P}}_{\mathrm{n}}\right\} \rightleftharpoons \left[\frac{({\mathrm{G}}_{1}+ {\mathrm{G}}_{\mathrm{n}})}{2}, \dots , {\mathrm{G}}_{2}, {\mathrm{G}}_{3}, \dots , {\mathrm{G}}_{\mathrm{n}}\right],\mathrm{ n}\in \mathrm{N}$$

### Functional annotations

For analyzing the targets of XFZYD at the functional level, Gene Ontology (GO) analysis were performed by clusterProfiler package of R software with *p*-values of 0.05 [21], Kyoto Encyclopedia of Genes and Genomes (KEGG) enrichment analyses were constructed base on KEGG database with *p*-values of 0.05 [22]. Graphs were created by ggplot2 package in R language (version 3.4.2).

### Experimental validation

#### Materials

Acacetin, quercetin, luteolin (≥ 98% purity by HPLC) was obtained from Jiangsu Yongjian Biotech Co., Ltd (Chengdu, China). Fetal bovine serum (FBS) and Dulbecco’s modifified Eagle’s medium (DMEM) were purchased from Gibco (Grand Island, USA). The mouse hippocampal HT22 cells were obtained from CHI SCIENTIFIC (Shanghai, China). Hypoxic bags were purchased from Mitsubishi Gas Chemical Company, Inc (Japanese). Cell Counting Kit-8 (CCK-8) was purchased from Dojindo Laboratories (Japanese).

### Cell culture and oxygen–glucose deprivation (ogd) treatment

HT22 cells were cultured in DMEM with 10% FBS, and incubated at 37 °C under 5% CO_2_. Hypoxic bags were used to perform OGD model according to the method which had been reported [23].

### Cell viability assay

HT22 cells (6 × 10^4^ cells/well) were plated in 96-well plates for 24 h, then the cells were exposed to OGD model with hypoxia for 12 h, followed by treatment with 40, 80, 120, 160 and 200 μM acacetin, quercetin, and luteolin, respectively. The acacetin, quercetin, and luteolin were dissolved in DMSO to reach a final concentration and further diluted to different concentrations using medium. After 24 h of incubation, 10 μL of CCK8 was added and incubated for a further 4 h. The plate reader was utilized to detect the absorbance at 450 nm.

### Statistical analysis

All data were expressed as mean ± SEM. The differences were analyzed by one-way ANOVA for multiple comparisons, and Student’s t test was utilized to compare the significance of differences between two groups. Results were considered as statistically significant if the p-value was < 0.05.

## Results

### The overview of our proposed integrated pharmacology model

**Fig. 1:**
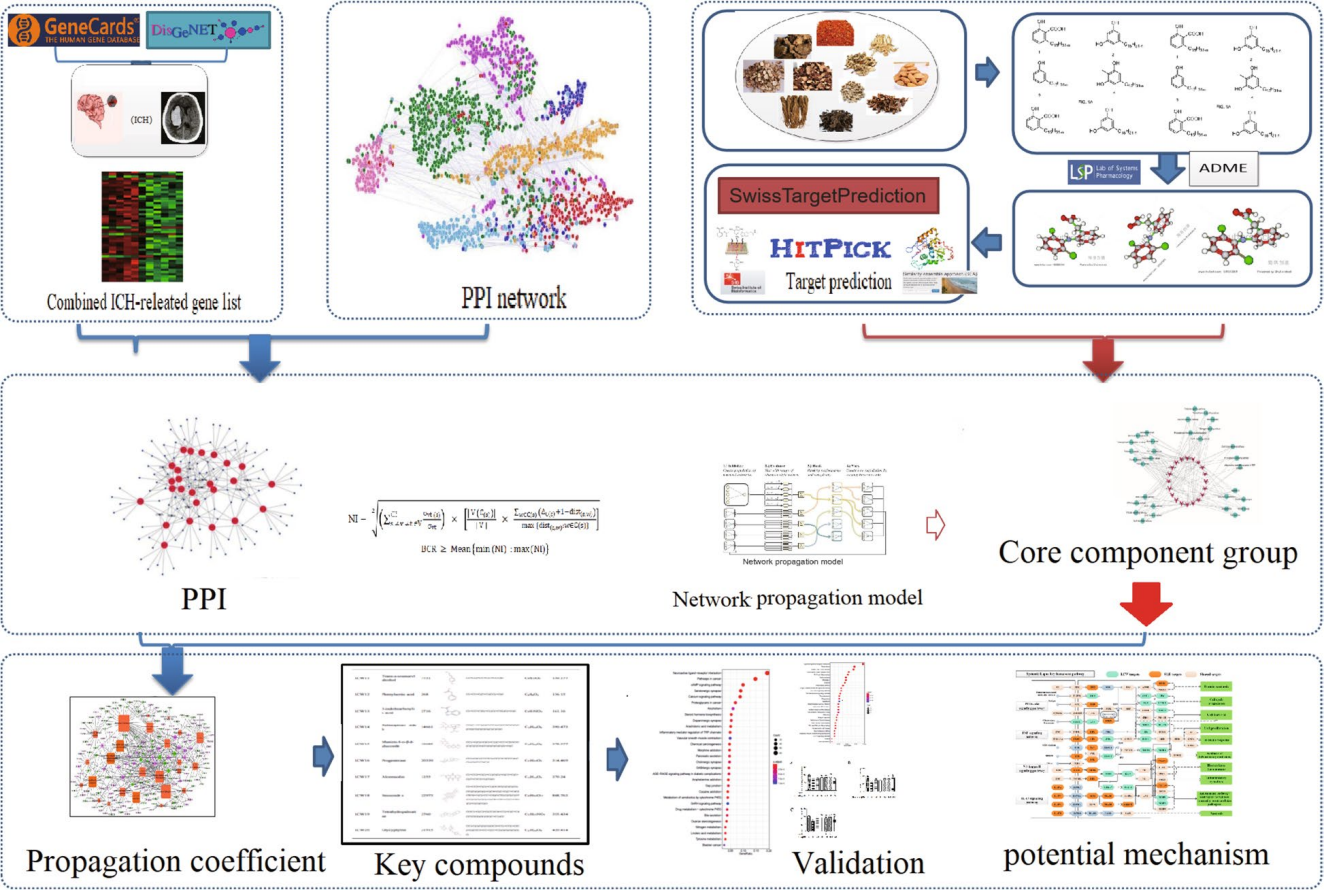
A schematic diagram of system pharmacology-based strategy to uncover the potential mechanism of Xue Fu Zhu Yu Decoction in treating Intracerebral Hemorrhage

Here, a novel integrated pharmacology module was designed to predict the CCG and depict the underlying mechanisms of XFZYD on ICH (Fig. 1). In the first step, all XFZYD compounds were extracted from database and literatures. Next, the active compounds were figured out from all compounds of XFZYD based on proposed ADME models. Then we use three online tools to predict the targets of these active compounds. The weighted pathogenic gene–gene interactions and active compounds-targets network were merged to build the high reliable effective space (HRES) for decoding the effective proteins. HRES was developed to determine the CCG. Finally, the CCG and their targets were used to figure out the underlying mechanisms of XFZYD in treating ICH.

### Construction of weighted pathogenic gene–gene interaction network for hypertensive cerebral hemorrhage

Weighted pathogenic gene–gene interaction network plays an important role in disease occurrence, drug response and other life activities. Constructing and analyzing weighted pathogenic gene–gene interaction network is the foundation in understanding the pathogenesis of ICH and can provide intervention strategies. To construct a weighted pathogenic gene–gene interaction network of ICH, PPI data were extracted and integrated from public databases, such as BioGRID, STRING, Dip, HPRD, Mint and Reactome. 863 genes were extracted from GeneCards related to ICH, and each gene has a correlation score. If the correlation score is higher than the average score of these genes, it will be retained and used to build a weighted pathogenic gene–gene interaction network. There are many topological properties that can be used to describe the network, among which the degree is one of the indicators to describe the importance of nodes in the network. Through network topology analysis, it is found that APP, SRC, MAPK1 and other genes have higher degrees. Zhu et al. found that amyloid precursor protein (APP) mutation can cause typical pathological changes of AD and perivascular amyloid deposition [24]. Fu et al. showed that NOTCH3 gene mutation may cause cerebral hemorrhage by changing the structure and function of cerebral small vessels [25]. Wang Lisong et al. found that the expression of c-myc is related to oxidative stress and neuronal apoptosis after intracerebral hemorrhage [26]. Qian Hong et al. found that EGFR expression increased in brain tissue around hematoma after intracerebral hemorrhage, and EGFR gene silencing was helpful to inhibit the activation of astrocytes in rats, and its mechanism might be related to blocking STAT3 phosphorylation [27]. These results indicate that these genes may play an important role in the pathogenesis of ICH.

### Chemical composition collection and composition analysis

Chemical identification is the critical step to clarify the material basis and action mechanism of prescription. In this study, the information of specific chemical compounds of XFZYD Chinese herbal medicines was obtained from literatures (Table 1). The results showed that the chemical compounds of herbs and the content of identified compounds provided experimental auxiliary chemical space for searching active compounds. The analysis of chemical constituents provides a reference for screening active constituents in XFZYD.

**Table 1:** The chemical information of the herbs in XFZYD from the literature

Herb method component concentration ref				
*Glycyrrhiza uralensis* Fisch. ex DC. (Gancao)	HPLC	Glycyrrhizin	97.49 mg/g	Chen et al. 2009 [28]
		Liquiritin	102.83 mg/g	
		Lsoliquritigenin	98.30 mg/g	
*Angelica sinensis* (Oliv.) Diels (Danggui)	HPLC	Ferulic acid	0.36 mg/g	Xie et al. 2007 [29]
		Coniferylferulate	6.11 mg/g	
		*Z*-lig ustilide	4.34 mg/g	
		*E*-ligustilide	0.23 mg/g	
		*Z*-3-butylidenephthalide	0.20 mg/g	
		*E*-3-butylidenephthalide	0.08 mg/g	
*Paeonia lactiflora* Pall. (Chishao)	RP- HPLC	Gallic acid	2.33 mg/g	Li et al. 2011 [30]
		Hydroxyl-paeoniflorin	1.89 mg/g	
		Catechin	0.03 mg/g	
		Albiflorin	4.44 mg/g	
		Paeoniflorin	4.81 mg/g	
		Benzoic acid	0.03 mg/g	
		1, 2, 3, 4, 6 -pentagalloylglucose	4.80 mg/g	
		Benzoyl -paeoniflorin	0.11 mg/g	
		Paeonol	0.07 mg/g	
*Citrus* × *aurantium* L.(Zhike)	HPLC	Naringenin	1.91 mg/g	Zhan et al. 2015 [31]
		Hesperetin	1.37 mg/g	
		Marmin	1.52 mg/g	
		6’,7’-Dihydroxy bergamot	2.96 mg/g	
		Citronella	2.90 mg/g	
		Orange peel	2.18 mg/g	
*Prunus alleghaniensis* Porter(Taoren)	HPLC	cytidine	0.43 mg/g	Li et al. 2013 [32]
		uridine	0.29 mg/g	
		guanosine	0.14 mg/g	
		adenosine	0.79 mg/g	
*Carthamus tinctorius* L.(Honoghua)	HPLC	Gallic acid	3.37 mg/g	Wu et al. 2019 [33]
		hydroxysafflor yellow A	97.92 mg/g	
		protocatechuic acid	34.19 mg/g	
		rutin	83.66 mg/g	
		quercetin	19.51 mg/g	
*Ligusticum chuanxiong* S.H.Qiu(Chuangxiong)	HPLC	ferulic acid	2.51 mg/g	Liu et al. 2014 [34]
		Ligusticum wallichii lactone	0.7 mg/g	
		Ligustilide	2.15 mg/g	
*Rehmannia glutinosa* (Gaertn.) DC.(Dihuang)	HPLC	Catalpol	2.3 mg/g	Wu.2016 [35]
		Ophiopogon glycoside	0.51 mg/g	
*Platycodon grandiflorus* (Jacq.) A.DC.(Jiegeng)	RP-HPLC	deapioplatycoside E	4.16 μg/mL	Wang et al. 2011 [36]
		platycoside E	10.57 μg/mL	
		platycodin D3	81.6 μg/mL	
*Achyranthes bidentata* Blume(Niuxi)	HPLC	25 R-inokosterone	0. 626 mg/g	Lang et al. 2019 [37]
		25S-inokosterone	0. 127 mg/g	
		ecdysterone	0. 274 mg/g	
		Ginsenoside R0	0. 701 mg/g	
		chikusetsu saponin IVa	1. 798 mg/g	
*Bupleurum chinense* DC.(Chaihu)	HPLC	Saikosaponin D	0.016 mg/g	Hu et al. 2011 [38]

### Selection of active compounds

A total of 716 compounds were found in XFZYD through systematic search of the compounds in the published database of 11 herbs. Each CHM prescription contains hundreds of compounds, only a few compounds possess suitable pharmacokinetic and pharmacology dynamics properties. In the present work, three published ADME models, OB, Caco-2 and DL, were used to pick out the active compounds. After ADME screening, due to its high concentration and high biological activity, some compounds which did not meet the three screening criteria were also kept as the active compounds. Finally, 179 active compounds were selected from 716 compounds (Table 2). Butyl-phthalide is a potential active compound of XFZYD. Tu et al. found that butyl-phthalide can significantly reduce the neurological deficit in rats with intracerebral hemorrhage, which may be ascribed to butyl-phthalide by up-regulating the expression of VEGF and Ang-2 protein and increasing the density of neovascularization around hematoma without increasing the risk of hematoma enlargement. Quercetin is the main compound of HL and has a wide range of biological activities. Xu et al. showed that early treatment with the best dose of quercetin could improve the brain injury after intracerebral hemorrhage model by inhibiting inflammatory reaction and reducing apoptosis [39], thus promoting the recovery of neural function. These results suggest that these compounds can treat ICH through multi-target synergistic mechanism.

**Table 2:** Components in XFZYD after ADME screening

Herb	MOL_ID	molecule_name	ob	caco2	drug-likeness
NX	MOL012461	28-norolean-17-en-3-ol	35.93	1.37	0.78
NX	MOL002714	baicalein	33.52	0.63	0.21
NX	MOL001454	berberine	36.86	1.24	0.78
NX	MOL000085	beta-daucosterol_qt	36.91	1.30	0.75
NX	MOL000358	beta-sitosterol	36.91	1.32	0.75
NX	MOL012505	bidentatoside,ii_qt	31.76	-0.01	0.59
NX	MOL001458	coptisine	30.67	1.21	0.86
NX	MOL002643	delta 7-stigmastenol	37.42	1.30	0.75
NX	MOL002897	epiberberine	43.09	1.17	0.78
NX	MOL003847	Inophyllum E	38.81	0.68	0.85
NX	MOL000422	kaempferol	41.88	0.26	0.24
NX	MOL000785	palmatine	64.60	1.33	0.65
NX	MOL001006	poriferasta-7,22E-dien-3beta-ol	42.98	1.45	0.76
NX	MOL000098	quercetin	46.43	0.05	0.28
NX	MOL004355	Spinasterol	42.98	1.44	0.76
NX	MOL000449	Stigmasterol	43.83	1.44	0.76
NX	MOL000173	wogonin	30.68	0.79	0.23
DG	MOL008259	2,6-di(phenyl)thiopyran-4-thione	69.13	1.74	0.15
DG	MOL000358	beta-sitosterol	36.91	1.32	0.75
DG	MOL000449	Stigmasterol	43.83	1.44	0.76
ZK	MOL000358	beta-sitosterol	36.91	1.32	0.75
ZK	MOL002341	Hesperetin	70.31	0.37	0.27
ZK	MOL013381	Marmin	38.23	0.14	0.31
ZK	MOL004328	naringenin	59.29	0.28	0.21
ZK	MOL005828	nobiletin	61.67	1.05	0.52
HH	MOL002694	4-[(E)-4-(3,5-dimethoxy-4-oxo-1-cyclohexa-2,5-dienylidene)but-2-enylidene]-2,6-dimethoxycyclohexa-2,5-dien-1-one	48.47	0.81	0.36
HH	MOL002712	6-Hydroxykaempferol	62.13	0.16	0.27
HH	MOL002719	6-Hydroxynaringenin	33.23	0.27	0.24
HH	MOL002757	7,8-dimethyl-1H-pyrimido[5,6-g]quinoxaline-2,4-dione	45.75	0.06	0.19
HH	MOL002714	baicalein	33.52	0.63	0.21
HH	MOL002773	beta-carotene	37.18	2.25	0.58
HH	MOL000358	beta-sitosterol	36.91	1.32	0.75
HH	MOL000953	CLR	37.87	1.43	0.68
HH	MOL000131	EIC	41.90	1.16	0.14
HH	MOL002680	Flavoxanthin	60.41	0.97	0.56
HH	MOL000422	kaempferol	41.88	0.26	0.24
HH	MOL002683	Ligla	45.01	1.20	0.15
HH	MOL002695	lignan	43.32	0.42	0.65
HH	MOL000432	linolenic acid	45.01	1.21	0.15
HH	MOL002698	lupeol-palmitate	33.98	1.52	0.32
HH	MOL000006	luteolin	36.16	0.19	0.25
HH	MOL001398	Methyllinolenate	46.15	1.48	0.17
HH	MOL000675	oleic acid	33.13	1.17	0.14
HH	MOL002706	Phytoene	39.56	2.22	0.50
HH	MOL002707	phytofluene	43.18	2.29	0.50
HH	MOL001771	poriferast-5-en-3beta-ol	36.91	1.45	0.75
HH	MOL002710	Pyrethrin II	48.36	0.53	0.35
HH	MOL002717	qt_carthamone	51.03	-0.31	0.20
HH	MOL002721	quercetagetin	45.01	-0.06	0.31
HH	MOL000098	quercetin	46.43	0.05	0.28
HH	MOL000449	Stigmasterol	43.83	1.44	0.76
CX	MOL000131	EIC	41.90	1.16	0.14
CX	MOL002203	Exceparl M-OL	31.90	1.39	0.16
CX	MOL001494	Mandenol	42.00	1.46	0.19
CX	MOL001641	METHYL LINOLEATE	41.93	1.44	0.17
CX	MOL002135	Myricanone	40.60	0.67	0.51
CX	MOL000675	oleic acid	33.13	1.17	0.14
CX	MOL002140	Perlolyrine	65.95	0.88	0.27
CX	MOL002151	senkyunone	47.66	1.15	0.24
CX	MOL000359	sitosterol	36.91	1.32	0.75
CX	MOL002157	wallichilide	42.31	0.82	0.71
GC	MOL004941	(2R)-7-hydroxy-2-(4-hydroxyphenyl)chroman-4-one	71.12	0.41	0.18
GC	MOL004805	(2S)-2-[4-hydroxy-3-(3-methylbut-2-enyl)phenyl]-8,8-dimethyl-2,3-dihydropyrano[2,3-f]chromen-4-one	31.79	1.00	0.72
GC	MOL004824	(2S)-6-(2,4-dihydroxyphenyl)-2-(2-hydroxypropan-2-yl)-4-methoxy-2,3-dihydrofuro[3,2-g]chromen-7-one	60.25	0.00	0.63
GC	MOL004945	(2S)-7-hydroxy-2-(4-hydroxyphenyl)-8-(3-methylbut-2-enyl)chroman-4-one	36.57	0.72	0.32
GC	MOL004815	(E)-1-(2,4-dihydroxyphenyl)-3-(2,2-dimethylchromen-6-yl)prop-2-en-1-one	39.62	0.66	0.35
GC	MOL004898	(E)-3-[3,4-dihydroxy-5-(3-methylbut-2-enyl)phenyl]-1-(2,4-dihydroxyphenyl)prop-2-en-1-one	46.27	0.41	0.31
GC	MOL004914	1,3-dihydroxy-8,9-dimethoxy-6-benzofurano[3,2-c]chromenone	62.90	0.40	0.53
GC	MOL004913	1,3-dihydroxy-9-methoxy-6-benzofurano[3,2-c]chromenone	48.14	0.48	0.43
GC	MOL005013	18α-hydroxyglycyrrhetic acid	41.16	-0.29	0.71
GC	MOL004959	1-Methoxyphaseollidin	69.98	1.01	0.64
GC	MOL004866	2-(3,4-dihydroxyphenyl)-5,7-dihydroxy-6-(3-methylbut-2-enyl)chromone	44.15	0.48	0.41
GC	MOL004978	2-[(3R)-8,8-dimethyl-3,4-dihydro-2H-pyrano[6,5-f]chromen-3-yl]-5-methoxyphenol	36.21	1.12	0.52
GC	MOL004849	3-(2,4-dihydroxyphenyl)-8-(1,1-dimethylprop-2-enyl)-7-hydroxy-5-methoxy-coumarin	59.62	0.40	0.43
GC	MOL004863	3-(3,4-dihydroxyphenyl)-5,7-dihydroxy-8-(3-methylbut-2-enyl)chromone	66.37	0.52	0.41
GC	MOL004905	3,22-Dihydroxy-11-oxo-delta(12)-oleanene-27-alpha-methoxycarbonyl-29-oic acid	34.32	-0.06	0.55
GC	MOL004966	3'-Hydroxy-4'-O-Methylglabridin	43.71	1.00	0.57
GC	MOL004974	3'-Methoxyglabridin	46.16	0.94	0.57
GC	MOL004864	5,7-dihydroxy-3-(4-methoxyphenyl)-8-(3-methylbut-2-enyl)chromone	30.49	0.90	0.41
GC	MOL004989	6-prenylated eriodictyol	39.22	0.40	0.41
GC	MOL004990	7,2',4'-trihydroxy-5-methoxy-3-arylcoumarin	83.71	0.24	0.27
GC	MOL004991	7-Acetoxy-2-methylisoflavone	38.92	0.74	0.26
GC	MOL003896	7-Methoxy-2-methyl isoflavone	42.56	1.16	0.20
GC	MOL004838	8-(6-hydroxy-2-benzofuranyl)-2,2-dimethyl-5-chromenol	58.44	1.00	0.38
GC	MOL004993	8-prenylated eriodictyol	53.79	0.43	0.40
GC	MOL000417	Calycosin	47.75	0.52	0.24
GC	MOL005020	dehydroglyasperins C	53.82	0.68	0.37
GC	MOL001792	DFV	32.76	0.51	0.18
GC	MOL004836	echinatin	66.58	0.38	0.17
GC	MOL004806	euchrenone	30.29	1.09	0.57
GC	MOL004915	Eurycarpin A	43.28	0.43	0.37
GC	MOL000392	formononetin	69.67	0.78	0.21
GC	MOL004996	gadelaidic acid	30.70	1.20	0.20
GC	MOL004856	Gancaonin A	51.08	0.80	0.40
GC	MOL004857	Gancaonin B	48.79	0.58	0.45
GC	MOL005000	Gancaonin G	60.44	0.78	0.39
GC	MOL005001	Gancaonin H	50.10	0.60	0.78
GC	MOL004910	Glabranin	52.90	0.97	0.31
GC	MOL004911	Glabrene	46.27	0.99	0.44
GC	MOL004908	Glabridin	53.25	0.97	0.47
GC	MOL004912	Glabrone	52.51	0.59	0.50
GC	MOL004828	Glepidotin A	44.72	0.79	0.35
GC	MOL004829	Glepidotin B	64.46	0.46	0.34
GC	MOL004808	glyasperin B	65.22	0.47	0.44
GC	MOL004811	Glyasperin C	45.56	0.71	0.40
GC	MOL004810	glyasperin F	75.84	0.43	0.54
GC	MOL005007	Glyasperins M	72.67	0.49	0.59
GC	MOL004879	Glycyrin	52.61	0.59	0.47
GC	MOL002311	Glycyrol	90.78	0.71	0.67
GC	MOL005008	Glycyrrhiza flavonol A	41.28	-0.09	0.60
GC	MOL004835	Glypallichalcone	61.60	0.76	0.19
GC	MOL004907	Glyzaglabrin	61.07	0.34	0.35
GC	MOL004957	HMO	38.37	0.79	0.21
GC	MOL004985	icos-5-enoic acid	30.70	1.22	0.20
GC	MOL001484	Inermine	75.18	0.89	0.54
GC	MOL004980	Inflacoumarin A	39.71	0.73	0.33
GC	MOL004948	Isoglycyrol	44.70	0.91	0.84
GC	MOL004949	Isolicoflavonol	45.17	0.54	0.42
GC	MOL001789	isoliquiritigenin	85.32	0.44	0.15
GC	MOL000354	isorhamnetin	49.60	0.31	0.31
GC	MOL004814	Isotrifoliol	31.94	0.53	0.42
GC	MOL000239	Jaranol	50.83	0.61	0.29
GC	MOL000422	kaempferol	41.88	0.26	0.24
GC	MOL004988	Kanzonol F	32.47	1.18	0.89
GC	MOL004820	kanzonols W	50.48	0.63	0.52
GC	MOL005003	Licoagrocarpin	58.81	1.23	0.58
GC	MOL005012	Licoagroisoflavone	57.28	0.71	0.49
GC	MOL000497	licochalcone a	40.79	0.82	0.29
GC	MOL004841	Licochalcone B	76.76	0.47	0.19
GC	MOL004848	licochalcone G	49.25	0.64	0.32
GC	MOL004882	Licocoumarone	33.21	0.84	0.36
GC	MOL004885	licoisoflavanone	52.47	0.39	0.54
GC	MOL004883	Licoisoflavone	41.61	0.37	0.42
GC	MOL004884	Licoisoflavone B	38.93	0.46	0.55
GC	MOL004904	licopyranocoumarin	80.36	0.13	0.65
GC	MOL004855	Licoricone	63.58	0.53	0.47
GC	MOL003656	Lupiwighteone	51.64	0.68	0.37
GC	MOL000211	Mairin	55.38	0.73	0.78
GC	MOL002565	Medicarpin	49.22	1.00	0.34
GC	MOL004328	naringenin	59.29	0.28	0.21
GC	MOL005016	Odoratin	49.95	0.42	0.30
GC	MOL005017	Phaseol	78.77	0.76	0.58
GC	MOL004833	Phaseolinisoflavan	32.01	1.01	0.45
GC	MOL002844	Pinocembrin	64.72	0.61	0.18
GC	MOL000098	quercetin	46.43	0.05	0.28
GC	MOL004961	Quercetin der	46.45	0.39	0.33
GC	MOL004827	Semilicoisoflavone B	48.78	0.45	0.55
GC	MOL004891	shinpterocarpin	80.30	1.10	0.73
GC	MOL004935	Sigmoidin-B	34.88	0.42	0.41
GC	MOL000359	sitosterol	36.91	1.32	0.75
GC	MOL000500	Vestitol	74.66	0.86	0.21
GC	MOL005018	Xambioona	54.85	1.09	0.87
TR	MOL001328	2,3-didehydro GA70	63.29	-0.27	0.50
TR	MOL000358	beta-sitosterol	36.91	1.32	0.75
TR	MOL000493	campesterol	37.58	1.31	0.71
TR	MOL000131	EIC	41.90	1.16	0.14
TR	MOL001339	GA119	76.36	-0.12	0.49
TR	MOL001340	GA120	84.85	0.38	0.45
TR	MOL001342	GA121-isolactone	72.70	-0.26	0.54
TR	MOL001343	GA122	64.79	-0.17	0.50
TR	MOL001344	GA122-isolactone	88.11	-0.18	0.54
TR	MOL001358	gibberellin 7	73.80	-0.18	0.50
TR	MOL001351	Gibberellin A44	101.61	-0.13	0.54
TR	MOL000296	hederagenin	36.91	1.32	0.75
TR	MOL001371	Populoside_qt	108.89	0.49	0.20
TR	MOL001323	Sitosterol alpha1	43.28	1.41	0.78
JG	MOL001689	acacetin	34.97	0.67	0.24
JG	MOL004580	cis-Dihydroquercetin	66.44	-0.34	0.27
JG	MOL000006	luteolin	36.16	0.19	0.25
JG	MOL004355	Spinasterol	42.98	1.44	0.76
CH	MOL004653	( +)-Anomalin	46.06	0.46	0.66
CH	MOL004598	3,5,6,7-tetramethoxy-2-(3,4,5-trimethoxyphenyl)chromone	31.97	0.75	0.59
CH	MOL004609	Areapillin	48.96	0.60	0.41
CH	MOL013187	Cubebin	57.13	0.47	0.64
CH	MOL000131	EIC	41.90	1.16	0.14
CH	MOL001789	isoliquiritigenin	85.32	0.44	0.15
CH	MOL000354	isorhamnetin	49.60	0.31	0.31
CH	MOL000422	kaempferol	41.88	0.26	0.24
CH	MOL001645	Linoleyl acetate	42.10	1.36	0.20
CH	MOL004624	Longikaurin A	47.72	0.08	0.53
CH	MOL004683	methyl (2E,4E)-octadeca-2,4-dienoate	38.77	1.45	0.17
CH	MOL004628	Octalupine	47.82	0.48	0.28
CH	MOL000675	oleic acid	33.13	1.17	0.14
CH	MOL000490	petunidin	30.05	0.16	0.31
CH	MOL000098	quercetin	46.43	0.05	0.28
CH	MOL004702	saikosaponin c_qt	30.50	0.03	0.63
CH	MOL004644	Sainfuran	79.91	0.90	0.23
CH	MOL000449	Stigmasterol	43.83	1.44	0.76
CH	MOL004648	Troxerutin	31.60	0.35	0.28
CH	MOL004718	α-spinasterol	42.98	1.28	0.76
CS	MOL000492	( +)-catechin	54.83	-0.03	0.24
CS	MOL006992	(2R,3R)-4-methoxyl-distylin	59.98	0.17	0.30
CS	MOL006994	1-o-beta-d-glucopyranosyl-8-o-benzoylpaeonisuffrone_qt	36.01	-0.03	0.30
CS	MOL006996	1-o-beta-d-glucopyranosylpaeonisuffrone_qt	65.08	-0.05	0.35
CS	MOL007008	4-ethyl-paeoniflorin_qt	56.87	-0.17	0.44
CS	MOL007012	4-o-methyl-paeoniflorin_qt	56.70	0.40	0.43
CS	MOL007018	9-ethyl-neo-paeoniaflorin A_qt	64.42	-0.01	0.30
CS	MOL007005	Albiflorin_qt	48.70	-0.38	0.33
CS	MOL002714	baicalein	33.52	0.63	0.21
CS	MOL000358	beta-sitosterol	36.91	1.32	0.75
CS	MOL000131	EIC	41.90	1.16	0.14
CS	MOL007022	evofolinB	64.74	0.00	0.22
CS	MOL001918	paeoniflorgenone	87.59	-0.09	0.37
CS	MOL007016	Paeoniflorigenone	65.33	-0.13	0.37
CS	MOL001925	paeoniflorin_qt	68.18	-0.34	0.40
CS	MOL000359	sitosterol	36.91	1.32	0.75
CS	MOL004355	Spinasterol	42.98	1.44	0.76
CS	MOL006999	stigmast-7-en-3-ol	37.42	1.32	0.75
CS	MOL000449	Stigmasterol	43.83	1.44	0.76
DH	MOL000131	EIC	41.90	1.16	0.14
DH	MOL003708	jioglutin D	39.02	-0.22	0.14
DH	MOL000359	sitosterol	36.91	1.32	0.75
DH	MOL000449	Stigmasterol	43.83	1.44	0.76

### Construction and analysis of c-t network

In order to explore the mechanism of XFZYD in treating ICH, a C-T network was constructed with 179 active compounds and 838 targets. There are 6559 relationships among compounds and their targets because of that some active compounds can relate to multiple targets. The average number of targets of per compound is 54.55. The C-T network showed that XFZYD had the characteristics of multi-targets and multi-compounds in treating ICH. In these compounds, MOL000675 (degree = 117) has the greatest number of targets, followed by MOL007022 (degree = 114), MOL000131 (degree = 111), MOL000098 (degree = 107) and MOL004996 (Degree = 106)), MOL001689 (degree = 104), MOL003896 (XFZYD 5, degree = 98), MOL004985 (XFZYD 2, degree = 98), MOL002203 (XFZYD 23, degree = 92). Most of these compounds are related to ICH apoptosis, inflammation and immune-related pathways. Parveen et al. found that ACER2 was involved in sphingomyelin metabolism, which could catalyze ceramide hydrolysis to produce sphingosine, and sphingosine was further phosphorylated to produce sphingosine monophosphate [40]. ACER2 can control cells by controlling the relative levels of ceramide, sphingosine and sphingosine monophosphate, thus playing a role in the process of cell proliferation, aging and apoptosis. Studies show that lysophosphatidic acid (LPA) is a kind of lysophosphatide with wide biological activity, which can cause a variety of growth factor-like reactions that regulate cell proliferation, migration and survival. The role of other compounds in ICH treatment has been described in the chapters "Shared compounds of XFZYD Chinese herbal medicine" and "Specific compounds of XFZYD Chinese herbal medicine". These results proved the important role of these compounds in treating ICH, and further confirmed the multi-compounds role of XFZYD in ICH treatment.

In the C-T network, the average target degree of different targets is 7.83. Most of the top 20 targets including MAPT, ALOX5AP, ESR2, ODC1, and ESR1, etc. which have the higher weights in the C-T network, are associated with immunity and inflammation and have been confirmed to be related to the pathogenesis of ICH, and may indicate the potential mechanism of XFZYD on ICH. For example, Markoula et al. found that ESR2 was significantly related to the risk of stroke [41]. ESR2 and COC exposure have synergistic effect in the occurrence of hemorrhagic stroke. Inflammation is the main pathogenic mechanism of atherosclerosis, and leukotriene is an important inflammatory mediator involved in the inflammatory reaction. Evans et al. showed that 5-lipoxygenase (5-LO) activator protein (FLAP) was encoded and synthesized by ALOX5AP gene, which was necessary for 5-LO activation and leukotriene synthesis [42]. Jiang found that ODC1 could be used as a common target to control inflammatory reaction and could exert its inhibitory effect on macrophage inflammatory reaction and inhibit ROS-induced macrophage apoptosis [43]. These results suggest that XFZYD can treat ICH through the synergistic effect of regulating inflammation and immune function, which further confirms the multi-target effect of XFZYD in the treatment of ICH.

The main targets of the active compounds are often involved in HIF-1 signaling pathway (hsa04066), VEGF signaling pathway (hsa04370), MAPK signaling pathway (hsa04010), Alzheimer disease (hsa05010) and PI3K-Akt signaling pathway (HSA 04,151). VEGF signaling pathway is very important for cell proliferation and growth. According to literatures, VEGF stimulates the expression level of the PI3K-Akt and MAPK-ERK proteins in the cerebral cortex of rats through phosphate Akt and activate MAPK-regulated ERK pathways, thus reducing the lesion volume of cerebral infarction and promoting angiogenesis in rats with cerebral infarction [44]. Studies have shown that PI3K is an important phosphatidylinositol kinase, which involve in cell growth and bone remodeling, and is an important anti-apoptosis regulator. Activation of AKT can induce the phosphorylation of lipid substrate and can promote downstream protein kinase B, which can contribute to cell proliferation and differentiation [45]. The results show that the optimization strategy of CHM prescription combining effective space with CI model is reliable, and the predicted CCG may play a therapeutic role by mediating various related ways.

### Selection and validation of effective proteins

Using weighted pathogenic gene–gene interactions and C-T network to construct compounds-targets-pathogenic genes (C-T-P) network. This network is consisting 2,932 nodes and 48,980 edges. Degree of each node can be used to evaluate the importance of nodes in the network. The node with the degree which is greater than the average degree of all nodes in the network is considered to play a key role in the network structure and can be regarded as a central node. According to this rule, the transmitted nodes and their edges are retained in the C-T-P network, and defined as the effective space. 445 effective proteins were identified from the effective space. There are three kinds of effective proteins in the effective space. The first category of making a table is the direct interaction between pathogenic genes and compounds targets. We defined this category as the basic effective compounds. The second category we defined as the targets specific interactions, which contain the interactions, only existed between the targets of compounds. The third category we defined as the pathogenic genes specific interactions, which mean the interactions linked the different pathogenic genes.

**Fig. 2:**
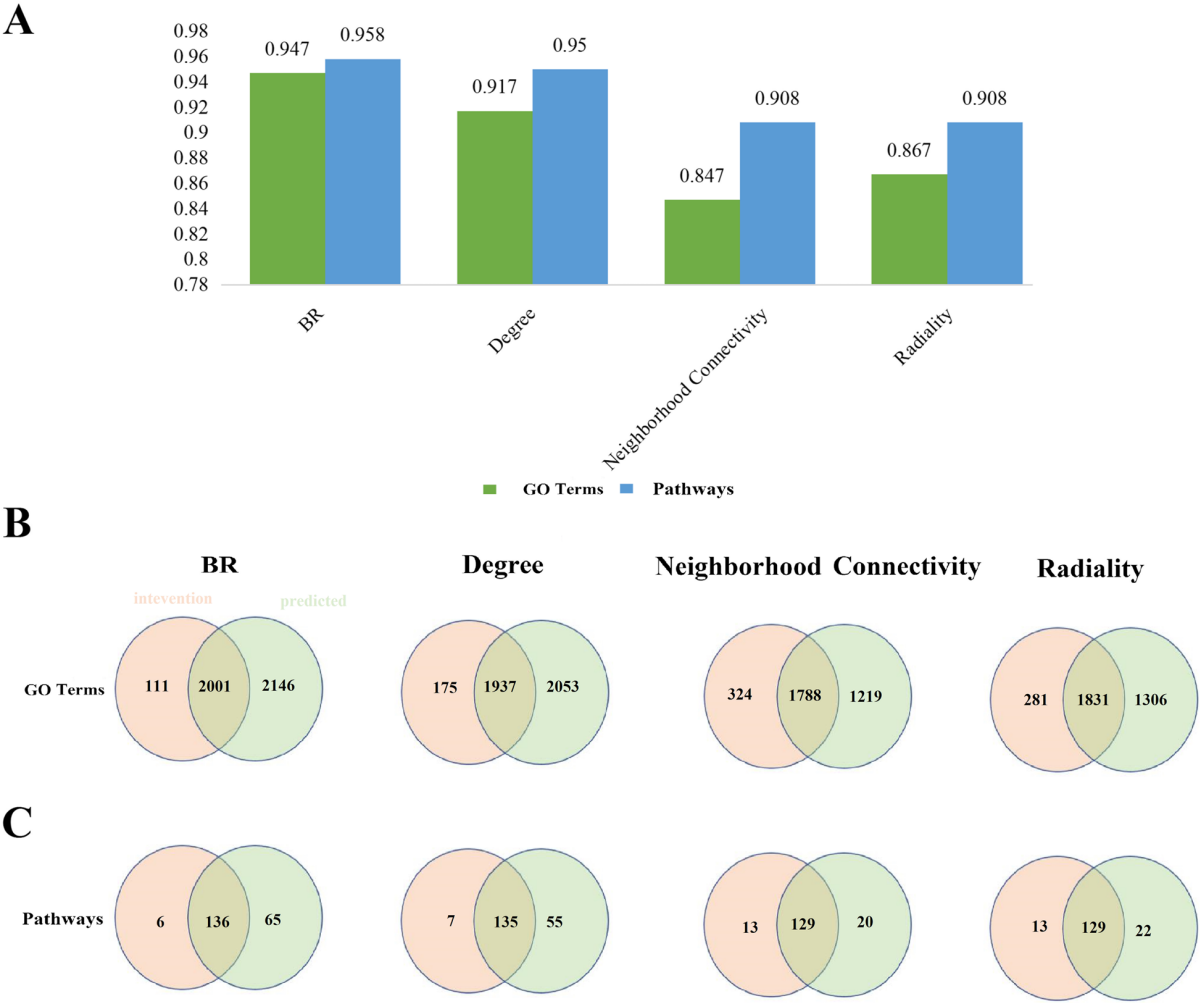
Compare our proposed model with other widely used models. **a** Venn diagrams display the number of overlapped GO terms of four models with main intervention GO terms. **b** Venn diagrams display the number of overlapped pathways of four models with intervention pathways, respectively. (**c**) Comparison of our model with other models on the intervention pathways and GO terms

In order to further prove the reliability and accuracy of our model, we compare our proposed node importance calculation method with other commonly used node importance calculation methods, including degree, betweenness centrality, and clustering coefficient. We used our model and these models to obtain respective coefficient proteins and used these effective proteins to perform pathway and GO enrichment analysis, respectively, and then check the percentages of effective protein-enriched pathways and GO terms in intervention pathways and intervention GO terms, respectively. The higher the percentage, the higher the reliability and accuracy of the model. Results show that the percentage of effective protein enriched pathways found in our model in intervention pathway and intervention GO term is significantly higher than that in the degree model, betweenness centrality model, and clustering coefficient model. These results show that compared with other node importance models, our model has higher accuracy and better functional coverage (Fig. 2).

**Fig. 3:**
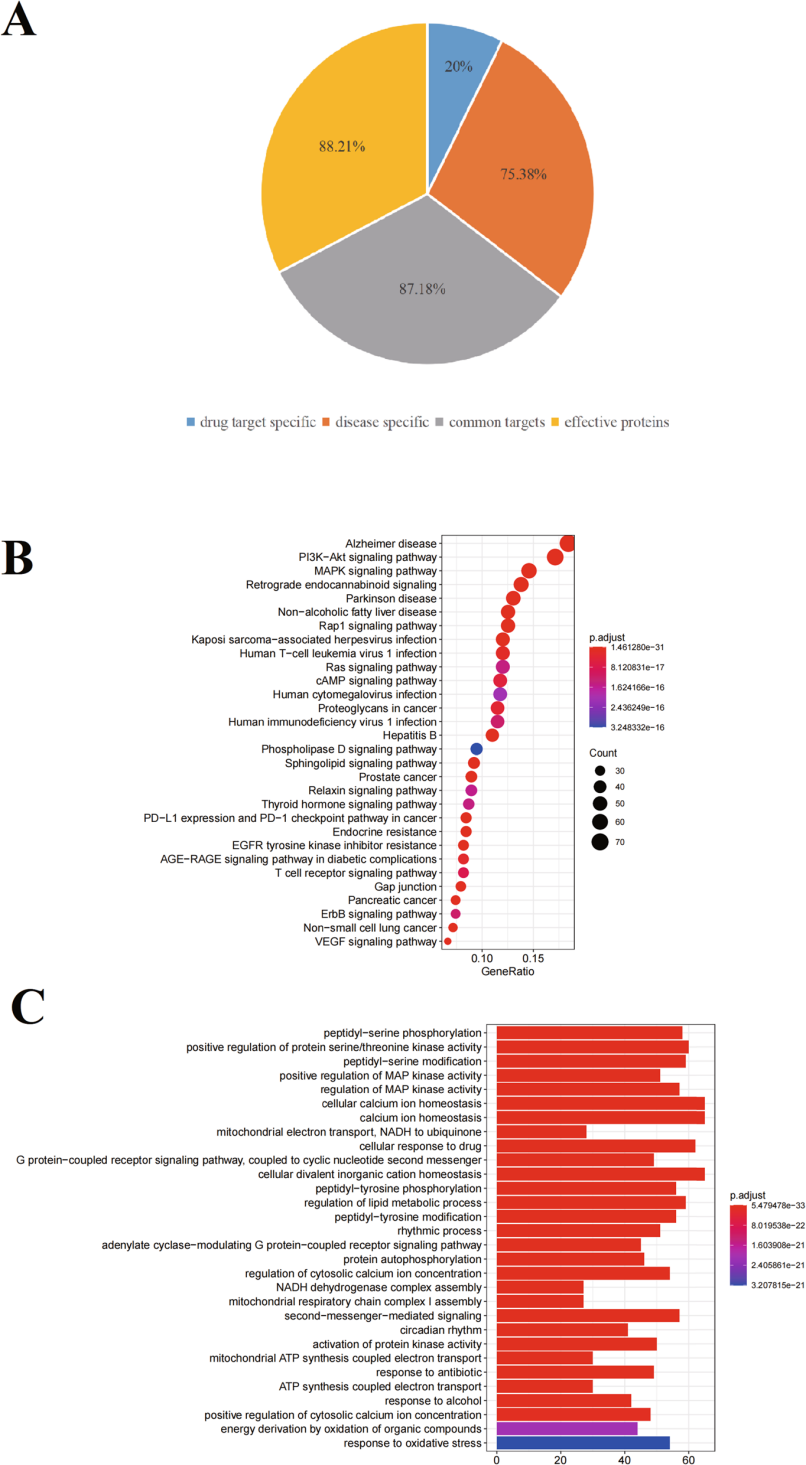
Effective space validation. The coverage rate of effective proteins, common targets, disease-specific targets, and drug-specific targets-enriched pathways compared with pathogenetic gene-enriched pathways of ICH (**A**). KEGG (**B**) and GO (**C**) enrichment processing of effective protein

We evaluated whether the targets of effective proteins screened from the effective space can cover the pathogenic genes of ICH at the functional level by enriched pathways. As result, targets of effective proteins have 172 enriched pathways (*p* < 0.05), while pathogenic genes can be enriched to 195 pathways (*p* < 0.05). It was found that effective protein enriched pathways covered 88.21% of pathogenic gene enriched pathways (Fig. 3). In addition, in order to evaluate whether the effective space can be replaced by basic effective interactions, pathogenic genes specific interactions or compounds targets specific interactions, so as to further optimize. We analyzed the paths of basic effective interactions; pathogenic genes specific interactions and compounds targets specific interactions. The results showed that the coverage rates of the three enriched pathways were 87.18%, 75.38% and 20.00%, respectively. These results validated the reliability and accuracy of our effective space selection method, and further proved that the effective protein selected in the effective space played leading roles in the pathogenesis of XFZYD. According to some literatures, PI3K/Akt/mTOR signaling pathway play important roles in cell proliferation and growth. The increase of Akt activity can reduce the expression of substrate p27kip1 in ICH [46]. Therefore, ICH lymphocytes accumulate towards apoptosis or proliferation in S phase and G2/M phase. Previous studies proved that the abnormal activation of PI3K/AKT signaling pathway may be involved in the pathogenesis of ICH in ICH patients by up-regulating CDKs and down-regulating p27Kip1 and p21WAF1/CIP1 [47]. These results of KEGG enrichments above and reports showed that XFZYD is related to the pathogenesis of ICH.

### Selection and verification of CCG

**Fig. 4:**
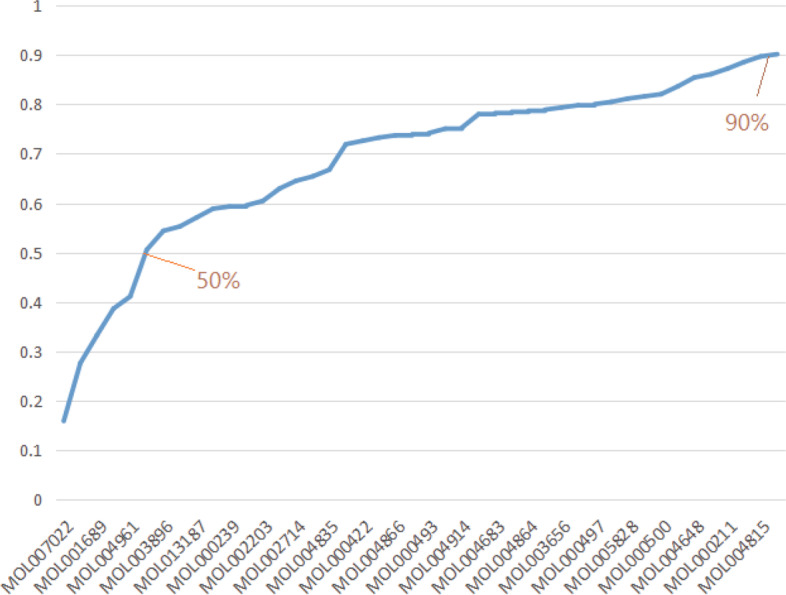
The accumulative CCG of effective compounds in effective space

Establish CI model, optimize effective compounds and get CCG, which lays a foundation for elucidating the underlying mechanism of XFZYD in treating ICH. According to the cumulative results of contributions, the top three compounds are EIC, quercetin and acacetin. The coverage rate of targets of wogonin and evofolin B to effective protein was 50.56%. For further analysis, 43 compounds can contribute to the target coverage of 90.11% of effective proteins and are selected as CCG (Fig. 4) The high targeted coverage rate of effective protein indicates that CCG may play a leading role in ICH treatment and produce joint effect.

**Fig. 5:**
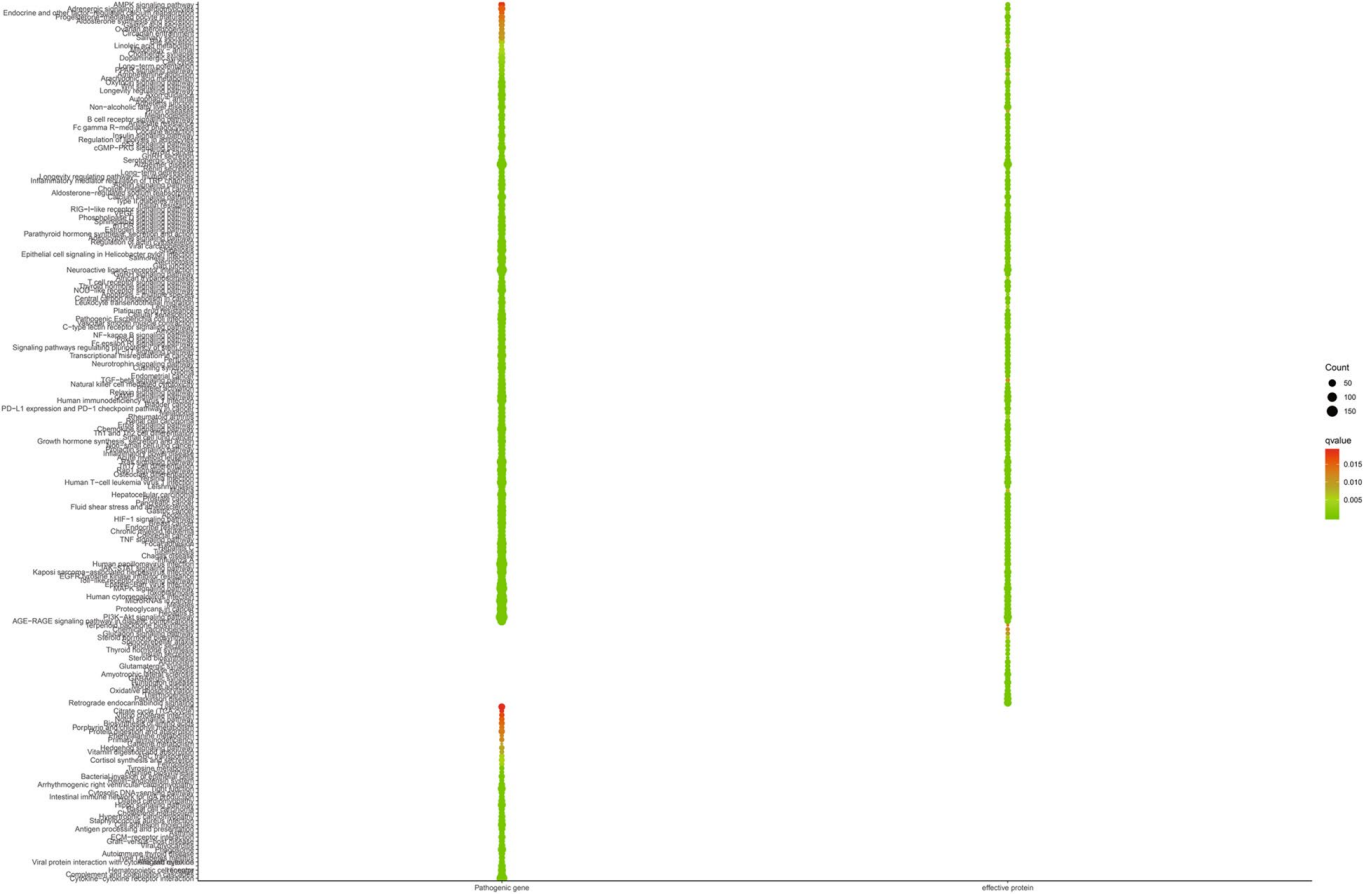
The functional similarity visualized between CCG targets and ICH pathogenic genes-enriched pathways

We analyzed the function of XFZYD in the treatment of ICH base on the KEGG enriched pathways of CCG targets and ICH pathogenic genes. As result, we obtained 173 CCG targeted enrichment pathways (*p* < 0.05) and 195 pathogenic gene enrichment pathways (*p* < 0.05). It was found that CCG targeted enrichment pathway covered 88.72% of pathogenic gene enrichment pathways (Fig. 5). The targets of CCG are mostly enriched in HIF-1 signaling pathway (hsa04066), VEGF signaling pathway (hsa04370), MAPK signaling pathway (hsa04010), Alzheimer disease (hsa05010) and PI3K-Akt signaling pathway (HSA 04,151). VEGF signaling pathway (hsa04370) is very important for cell proliferation and growth. The results show that the optimization strategy of CHM prescription combining optimization space with CI model is reliable, and the predicted CCG may play a therapeutic role by mediating various related ways. In order to verify whether our predicted CCG can represent all C-T network of XFZYD, two strategies are used to verify the reliability and accuracy of CCG. The first is based on the coverage of CCG pathogenic genes, which is defined as the percentage of the number of pathogenic genes in the network and C-T network; the second is based on network topology. Evaluate whether the number of CCG pathogenic genes is nearly equal to the number of ICH pathogenic genes in these 2 strategies, we found that C-T network contains 333 pathogenic genes. There are 298 pathogenic genes in CCG gene sequence. Compared with C-T network, the number of pathogenic genes in this network reached 89.49%, which confirmed that the predicted CCG had higher disease coverage rate. These results indicate that CCG has a high degree of agreement with C-T network in the number and coverage of pathogenic genes.

### Function analysis of CCG targets

**Fig. 6:**
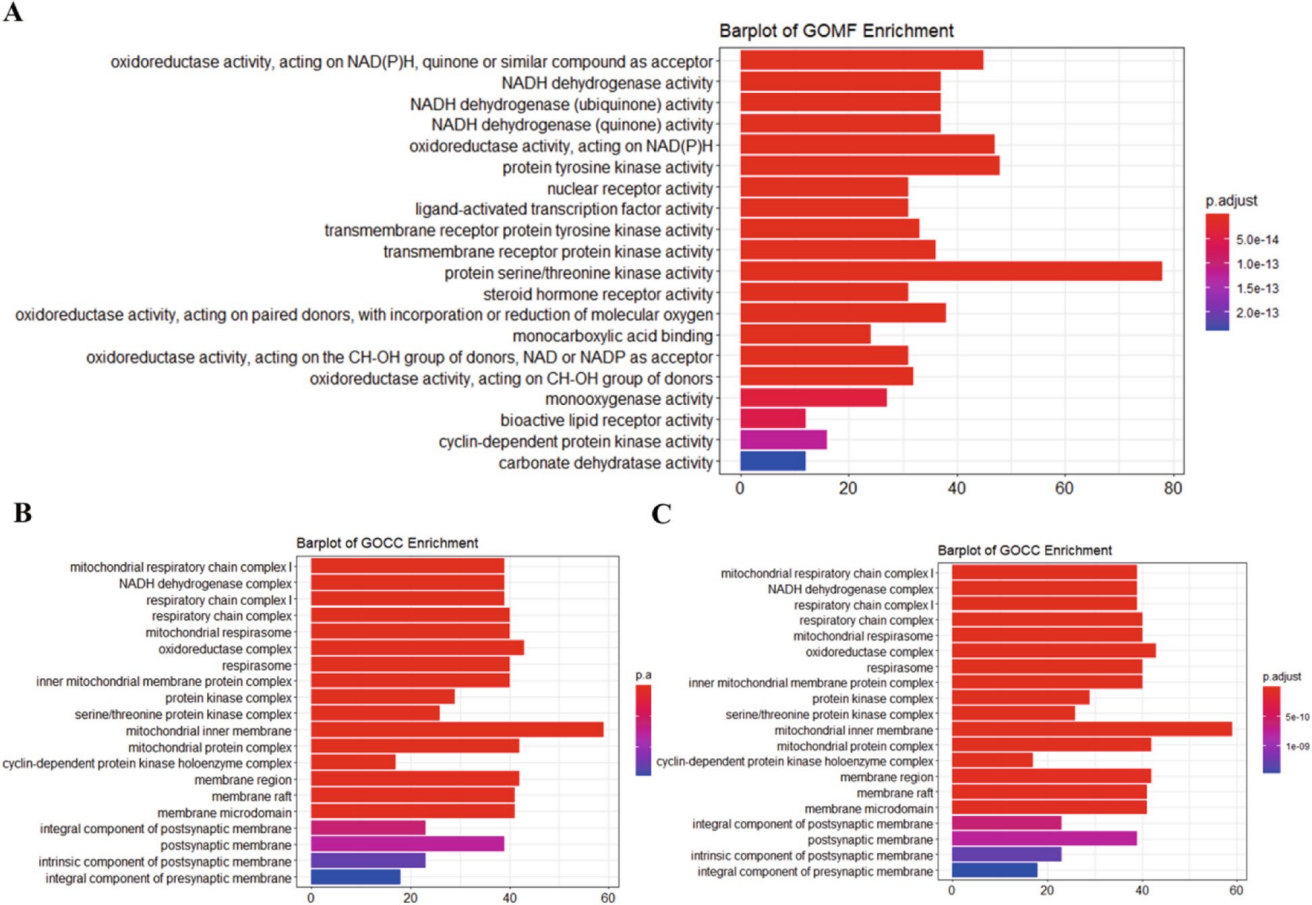
GO enrichment processing of CCG targets

GO enrichment analysis was performed using the R software clusterProfiler package to identify the biological functions of the main targets with *p* values < 0.05. To further profile the combined effects of XFZYT, all targets that interacted with CFCG in XFZYT were enriched by GO enrichment analysis (Fig. 6).

GO analysis showed that the regulatory targets of XFZYT were abundantly expressed in biological processes related to inflammatory response, oxidative stress and apoptosis. For example, leukocyte activation involved in inflammatory responses (GO:0,002,758), the production of molecular mediators involved in inflammatory responses (GO:0,070,498), and inflammatory responses to antigen stimulation (GO:0,031,349). These results confirmed that XFZYT could treat ICH by reducing inflammatory reaction, reducing oxidative stress and inhibiting apoptosis.

### Pathway enrichment analysis of CCG

**Fig. 7:**
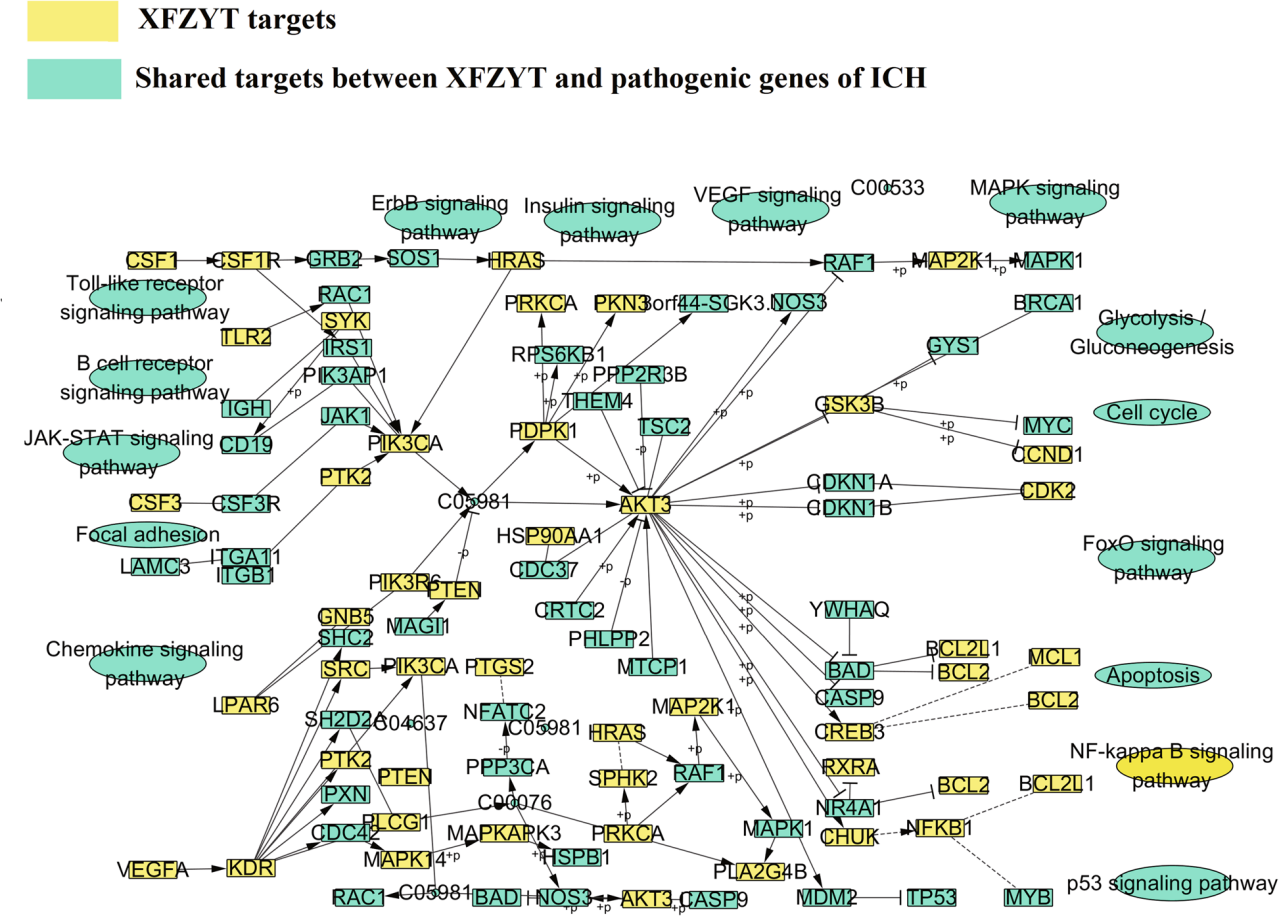
Distribution of targets of XFZYT on the compressed ICH pathway

ICH-related pathways can be decomposed into functional modules such as apoptosis, inflammation, and oxidative stress. Increasing evidences confirmed that the PI3K-Akt signaling pathway (hsa04151), JAK-STAT Signaling pathway (hsa04630), NF-kappa B signaling pathway (hsa04064), and IL-17 signaling pathway (hsa04657) respond to these functional modules. For example, PI3K-Akt signaling pathway (hsa04151) has been reported to be involved in the inhibition of apoptosis, cell proliferation, and expression of inflammatory cytokines. JAK-STAT signaling pathway (hsa04630) is involved in pathophysiological changes in damaged peripheral brain tissue after cerebral hemorrhage, leading to brain tissue edema. NF-kappa B signaling pathway (hsa04064) is a general name of a family of transcription factors that function as dimers to regulate genes of immunity, inflammation and cell survival. IL-17 signaling pathway (hsa04657), a subset of cytokines composed of IL-17A-F, plays an important role in acute and chronic inflammatory responses. For exploring the mechanism of XFZYT in the treatment of ICH at the system level, we constructed a comprehensive signaling pathway using two important molecular pathways (Fig. 7), the PI3K-Akt signaling pathway and the MAPK signaling pathway. This pathway plays an important role in the treatment of cerebral hemorrhage. We treated the first three columns as upstream locations of the integrated pathway, and the remaining columns as downstream locations of the integrated pathway. Among them, PI3K-Akt signaling pathway (hsa04151) is one of the main pathways for XFZYT to treat ICH. XFZYT regulated 12 targets upstream of the PI3K-Akt signaling pathway (hsa04151), such as KDR, VEGFA, and PTK2 and 35 targets downstream, such as Akt3 and CHUK. Downstream targets account for more than 70%. XFZYT may activate the downstream PI3K and AKT proteins through the upstream KDR, resulting in the cascade amplification of downstream PIK3CA, Akt3, and GSK3B, which is closely related to ICH cell proliferation and protein synthesis. Most of the targets of XFZYT in the regulation of PI3K-Akt signaling pathway are located in the downstream of this pathway, such as Akt3 and PIK3CA. After the PI3K/AKT signaling pathway is activated, it can regulate the apoptosis of other downstream target proteins to exert biological effects, playing a key role in the treatment of ICH.

### Experimental validation in *vitro*

**Fig. 8:**
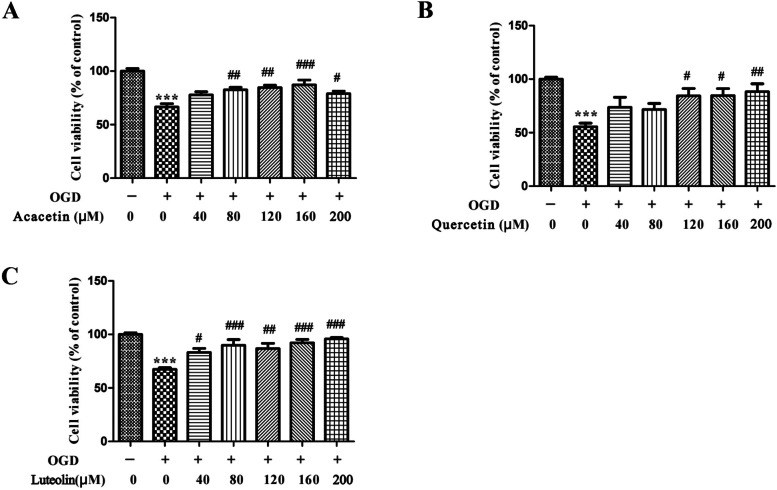
Effects of acacetin (**A**), quercetin (**B**), and luteolin (**C**) on cell viabilities. ***p < 0.001 compared with control group. #*p* < 0.05, #*p* < 0.05, ###*p* < 0.001 compared with the OGD group

To validate the reliability of our strategy, the important CCG predicted were experimentally validated on OGD model. The acacetin, quercetin, and luteolin with the highest gene coverage were selected for experimental verification. OGD model is a common model of cerebral hemorrhage and cerebral infarction in vitro by simulating the situation of stroke. Compared with control group, the cell viability of OGD groups were significantly reduced. Compared with control group, the effect of cell viability was significantly decreased by 33.48% in the hypoxia treated cells. However, Acacetin (80, 120, 160 and 200 μM) markedly increased the cell viability level by 16.00%, 18.10%, 20.73% and 12.42% (Fig. 8A). Compared with control group, the effect of cell viability was significantly decreased by 44.38% in the hypoxia treated cells. However, quercetin (120, 160 and 200 μM) markedly increased the cell viability level by 28.77%, 29.40% and 32.66% (Fig. 8B). Compared with control group, the effect of cell viability was significantly decreased by 32.59% in the hypoxia treated cells. However, Luteolin (40, 80, 120, 160 and 200 μM) markedly increased the cell viability level by 15.82%, 22.52%, 19.42%, 24.84% and 28.35% (Fig. 8C). The above results demonstrated that acacetin, quercetin and luteolin possessed protect effect in hypoxia treated HT22 cells.

## Discussions

In the process of treating complex diseases, Chinese medicine prescriptions usually exert their functional effects according to compatibility principles. Chinese medicinal prescriptions composed of different herbal medicines often contain hundreds of chemical compounds. Some of these chemicals have a synergistic therapeutic effect, while some may have an antagonistic therapeutic effect. How to figure out the synergistic or antagonistic effects of these chemical compounds and optimize the core therapeutic chemical compounds is the main purpose of prescription optimization.

In addition, in the process of treating diseases, the action form of prescriptions in TCM is relatively complex, and usually acts in the form of multi-compounds, multi-targets and multi-pathways, which constitutes a multi-dimensional regulatory network. How to find the core functional compound groups in these regulatory networks and analyze the mechanism is one of the key ways for us to understand the mechanism of prescriptions in the treatment of complex diseases.

Node importance is one of the most important ways to depict the network. In this research, we designed a new node importance calculation method and validated the accuracy and reliability. Based on this new node importance calculation method, optimization space was constructed. Potential effective proteins were screened out based on the optimization space, and then the key constituent groups corresponding to the effective proteins were obtained using the reverse programming algorithm. We analyzed the enrichment pathways of these CCG and found that the target enrichment pathways of these CCG accounted for 90.11% of the enrichment pathways of pathogenic genes. These results fully proved the accuracy and reliability of our computational model for screening key compound groups based on the optimization space. Our proposed screening strategy consists of two levels of design. The first level of design is to construct the optimization space based on the node importance calculation method, and verify that the information of disease-related genes will be preserved as much as possible in this step as the core protein or functional protein in the optimization space. In the second step, potential CCG are reversely screened based on the core proteins or functional proteins, and further pathway enrichment analysis is performed on the functional compound group for verification. The results of this dual model and verification fully demonstrate the reliability of our integrated analysis model, and provide methodological reference for other prescription optimization in TCM.

## Data Availability

The datasets used or analysed during the current study are available from the corresponding author. Similarity Ensemble Approach: https://sea.bkslab.org/ GeneCards database: https://www.genecards.org/ STRING: https://cn.string-db.org/ BioGRID: https://thebiogrid.org/ HPRD: http://www.hprd.org/.

## Abbreviations

CI: Contribution index
DL: Drug-likeness
GO: Gene Ontology
CCG: Key group of effective compounds
KEGG: Kyoto Encyclopedia of Genes and Genomes
XFZYD: Xue Fu Zhu Yu Decoction
OB: Oral bioavailability
PPI: Protein-protein interactions
ICH: Hypertensive Cerebral Hemorrhage
CHM: Traditional Chinese medicine
